# Hemodynamic Activity and Connectivity of the Prefrontal Cortex by Using Functional Near-Infrared Spectroscopy during Color-Word Interference Test in Korean and English Language

**DOI:** 10.3390/brainsci10080484

**Published:** 2020-07-27

**Authors:** Gihyoun Lee, Ji-Su Park, Mezie Laurence B. Ortiz, Jun-Yong Hong, Seung-Ho Paik, Seung Hyun Lee, Beop Min Kim, Young-Jin Jung

**Affiliations:** 1 Department of Physical and Rehabilitation Medicine, Center for Prevention and Rehabilitation, Heart Vascular Stroke Institute, Samsung Medical Center, Sungkyunkwan University School of Medicine, Seoul 6351, Korea; ghlee@skku.edu; 2 Advanced Human Resource Development Project Group for Health Care in Aging Friendly Industry Dongseo University, Busan 47011, Korea; jisu627@hanmail.net; 3 College of Medical Imaging and Therapy, De La Salle Medical and Health Sciences Institute, Cavite 4114, Philippines; mbortiz@dlshsi.edu.ph; 4 Department of Radiological Science, Dongseo University, Busan 47011, Korea; hohohong6161@hanmail.net; 5 Department of Bio-Convergence Engineering, Korea University, Seoul 6351, Korea; paikjang@korea.ac.kr (S.-H.P.); aksska82@korea.ac.kr (S.H.L.); bmk515@korea.ac.kr (B.M.K.)

**Keywords:** hemodynamic connectivity, brain network, hemodynamic response, functional near-infrared spectroscopy, color-word interference

## Abstract

In daily living, people are challenged to focus on their goal while eliminating interferences. Specifically, this study investigated the pre-frontal cortex (PFC) activity while attention control was tested using the self-made color-word interference test (CWIT) with a functional near-infrared spectroscopy device (fNIRS). Among 11 healthy Korean university students, overall the highest scores were obtained in the congruent Korean condition 1 (CKC-1) and had the least vascular response (VR) as opposed to the incongruent Korean condition 2 (IKC-2). The individual’s automatic reading response caused less brain activation while IKC-2 involves color suppression. Across the three trials per each condition, no significant differences (SD) in scores and in VR since there was no intervention did. Meanwhile, SD was observed between CKC-1 and English Congruent Condition 3 (ECC-3) across trials. However, SD was only observed on the third trial of VR. In the connectivity analysis, right and left PFC are activated on ECC-3. In CKC-1 and IKC-2, encompassing dorsomedial and dorsolateral although CKC-1 has less connection and connectivity due to less brain activation as compared. Therefore, aside from VR, brain connectivity could be identified non-invasively using fNIRS without ionizing radiation and at low-cost.

## 1. Introduction

In daily living, it is inevitable for an individual to be in a situation where distractions are not encountered. Thus, good control of focus is indeed important to achieve a particular goal in mind while eliminating interferences. Anatomically, it is the pre-frontal cortex (PFC), a part of the brain that is responsible for exercising executive function during this kind of situation [1]. Executive functions or also known as cognitive or executive control are defined as the set of cognitive processes needed by an individual when focus and attention are required or simply when the time to think is needed before doing a particular action [2]. Nowadays, many non-invasive modalities are being used to measure brain activity, however, one of those is the functional near-infrared spectroscopy (fNIRS) that is utilized in this study. It is considered as non-invasive, non-ionizing, easy to use, and low-cost that could improve patient’s quality of life [3]. Aside from those characteristics, Schroeter and colleagues [4] also added that it is flexible, portable, and can determine biochemical specificity as compared with other imaging that can be used to patients and children. Using the fNIRS device, this study aims to determine the hemodynamic activity or the vascular response (VR) of the brain when attention control is tested using the self-made color-word interference test (self-made CWIT) which is a modification of classic Stroop task.

Previous studies focused on within- and between-language differences, and not on the differences present between two sets of tests with a different language which was investigated in this study. In particular, this study would like to determine the mean scores and overall mean scores obtained in each condition per trial, if there are any significant differences per trial in each condition, between congruent and incongruent conditions using their native language, and lastly, between congruent conditions of subjects’ native and second languages. Moreover, the top 10 networks among fNIRS channels during the three conditions using the phase-locking value (PLV) will also be identified. It is hypothesized in this study that there are significant differences in mean scores and overall scores between self-made CWIT and VR, among trials per condition, trials between congruent conditions, and trials between congruent and incongruent conditions at a *p*-value of <0.05.

Specifically, the classic Stroop task [5] is among the popular ones that are used to test the attention control of an individual that has three conditions, such as the neutral condition, the congruent condition, and the incongruent condition. In the neutral condition, the word shown has no relevance to the ink color (e.g., the word “xxx” with an ink color of blue). The congruent condition means that the color word in itself has the same color as its ink color when shown (e.g., the word “blue” shown by using a blue ink color) and lastly, the incongruent condition in which the color word in itself does not represent its ink color when shown (e.g., the word “blue” shown by using a red ink color). In this task, the neutral condition versus the congruent condition displays facilitation, while the incongruent condition versus the congruent condition displays interference [6,7].

Treisman [8], proposed that the inability to focus attention in one dimension among several dimensions is what triggers the interference. Since interference is present in the incongruent task, it is said that the incongruent task is more difficult as compared with the congruent task. To enable us to understand the ability to counteract this interference and to execute the needed response during the Stroop task, various theories were developed. One of those is the theory of response competition by Eriksen and Eriksen [9], stating that the two different responses, such as the correct and incorrect response compete for the single response channel which necessitates the inhibition of the incorrect response. In the case of an incongruent task, the word response must be suppressed, leaving the color response, to make a correct response. Another theory is the theory of automaticity by Stirling [10]. This theory supports the idea of prolonged exposure to the same stimulus leads to the production of automatic response wherein attention control will no longer be needed. This implies that repeated exposure of a subject to certain stimuli will make the execution of a certain task easier.

In addition to the following mentioned above, there are studies conducted in the past to determine brain activity using the Stroop task. A study among Nepalese medical students showed that it took a long time for the subjects to read incongruent Stroop tasks and 60% of them made mistakes in reading, unlike in the congruent task where none of them committed a mistake [11]. In support of this, another study written by Hiroyasu and colleagues [12] presented that the number of correct responses among healthy young subjects was higher during congruent color-word Stroop task as compared in the incongruent color-word Stroop task. Aside from this, researches in the past focused on the determination of brain activity during the Stroop task. The specific parts of the brain that were activated are identified and most of them established the role of the prefrontal cortex (PFC) in attention control in the presence of interference [1,4,12,13,14,15,16] which is also the focus in this study with the use of the fNIRS device.

Among healthy subjects, the anterior cingulate cortex (ACC) that is connected with PFC [4,13] has shown activation in fMRI studies during the incongruent task of the Stroop task. Also, greater activation of the inferior frontal region [13] was observed when the subjects underwent the incongruent Stroop task, with more activation noted on the left inferior frontal region than the right inferior frontal region than during congruent task [12]. In the study of Lague–Beauvais and colleagues [15], the bilateral ventral prefrontal cortex (VLPFC) and dorsolateral prefrontal cortex (DLPFC), particularly the left posterior DLPFC and right anterior DLPFC has also shown activation during interference condition among older adults, as opposed to younger adults showing no significant activation of the prefrontal cortex.

Other studies demonstrated a more general activation of the lateral prefrontal cortex [4] and left dorsolateral regions [14]. Studies were also made regarding the use of the Stroop task in Chinese dyslexic children and those with prefrontal lesions. Sun and colleagues [4] demonstrated that the bilateral PFC activated upon doing the difficult color task for healthy groups, while the dyslexic group showing no significant PFC activation. Their findings suggest that conflict resolution among Chinese dyslexic children is not functional, as shown by the deactivation of their PFC. Meanwhile, Vendrell and colleagues [16] focused on determining if there are differences among healthy subjects and patients with prefrontal lesions when doing the Stroop task. It was found out that patients with lesions in the right lateral PFC made errors than healthy subjects, which suggests that the right lateral PFC is mostly associated with Stroop task errors. Also, patients who have undergone left lobectomy were able to perform the Stroop task normally. More importantly, the role of the right PFC has been noted in sustained attention, contradicting most of the studies indicating that the left PFC has a role in the inhibition of responses.

Likewise, in the previous studies, different modifications of the Stroop task have been made to get the desired outcome, which was also done by the authors of this study in which self-made CWIT using the native and second language of the students were employed. [7]. Previously the classic and modified Stroop condition were administered using different languages.

In detail, the study of Fang, Tzeng, and Alva [17] tested the Chinese-English (with Chinese as their native language) and Spanish-English (with Spanish as their native language) students using color-word incongruent Stroop task in Chinese, English, and Spanish language. It was found out that the response time in the Stroop test displayed in their second language is longer (1.431 s vs. 1.221 s for Chinese-English group; 1.169 s vs. 1.110 s for Spanish-English group). Furthermore, the prefrontal cortex was found out to have a role in encountering two different languages.

Moreover, in the study of Bialystok and colleagues [18] concerning the control in the cognitive aspect and lexical access involving younger and the older individuals doing bilingualism, it was concluded that in the congruent tasks, individuals can depend on the inadvertent reading reaction. However, Bugg and colleagues [19] stressed that for an individual to respond correctly in the incongruent tasks, the conflict existing between automatic reading and color-naming response must be resolved.

Also, in the study of Marian and colleagues [20], it was mentioned that in comparison with an individual who only speaks one language, those who can do bilingualism proficiently are expected to encounter two types of interference such as the color-naming and the language. Thus, Stroop effect requires inhibiting the dominant automatic reading response and language suppression that are not used in the specific task.

Though the study of Kim and colleagues [21] focused on verbal working memory (WM), the study discussed the active and relevant type of memory involved during language comprehension and problem-solving using the native language (Korean) and second language (English) for a short period. As proposed, the system of the brain temporarily stored and manipulate the essential information needed for several cognitive operations and one of those is linguistic. Also, it was proposed that there are three components for the WM, such as the central executive that is responsible for attention control and flow of information; second is the visuospatial sketch pad in which the visual images were set and maintained, and lastly is the phonological loop which the storage and rehearsal of verbal information happened. The findings of their study suggested that the right dorsolateral PFC is activated during the use of both languages.

In the study of Schroeter and colleagues [4], the effect of the Stroop task in the hemodynamic response was specifically investigated and it was concluded in their study that in the incongruent tasks, the response is stronger as compared with the congruent task. Hemodynamic response means oxygenated- and total-hemoglobin increased while a decrease in deoxygenated hemoglobin happened which is the same definition in the present study.

As compared with the above-mentioned studies, the present study specifically investigated the hemodynamic response of the prefrontal cortex of the brain during the congruent and incongruent self-made CWIT among healthy Korean university students using their native and second language.

## 2. Material and Methods

### 2.1. Subjects

Eleven healthy Korean native male young adults (19–25 years of age) were recruited through an institutional review board-approved procedure. The selection of subjects according to age and sex were not strict since the Stroop interference is the same regardless of age and sex [7], however, male subjects were preferred in this study since they have shorter hair compared to female subjects. This is to avoid hair interference between optodes and scalp contact for better signals acquisition.

All of the subjects have normal color vision, normal to corrected vision, and had no personal and family history of any neurological or psychological problems. Moreover, it was assured that the subjects know the English translation of colors by an assessment during the interview among subjects. They are known to be bilingual in which the English language is what they used to speak aside from their native language. All participants have learned their Korean language since birth and have been using it as their native language for more than twenty years. Also, they have studied English as their second language for more than ten years. They are selected from the same major and same university.

After explaining the procedure, informed consent was presented and collected from the subjects to ensure they agreed to participate in the study. After, a financial benefit was given individually as an appreciation for participating amounting to thirty-thousand Korean Won.

### 2.2. Color-Word Interference Test Material and Procedure

**Figure 1:**
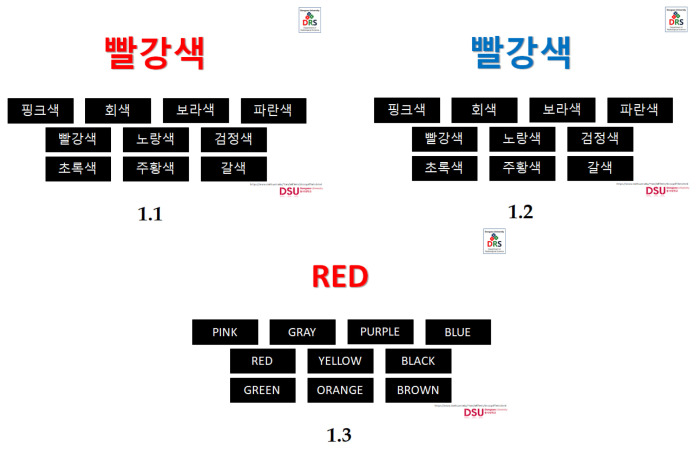
The self-made CWIT. (1.1) CKC-1: In the Korean language; the color word is the same as the ink color (빨강색 = red), (1.2) IKC-2: In the Korean language; the color word is different with the ink color, and (1.3) CEC-3: In English language; the color word is the same with the ink color.

The self-made color-word interference test was composed of four conditions originally, such as the congruent task in Korean language (ink color and word color in each item were the same, named CKC-1), the incongruent task in Korean language (ink color and word color in each item were different, named IKC-2), the congruent task in English language (named CEC-3) and the incongruent task in English language (named IEC-4). Each condition had 45 items, divided into three trials (15 items each trial). The conditions were CKC-1, IKC-2, CEC-3, and IEC-4. Resting time of 30 s before each trial was also observed. During the conduct of the experiment, the conditions were presented in a Microsoft PowerPoint file with an automatic timer set for each slide (see Figure 1). The duration of two seconds for each item, 30 s per trial, and one and a half minutes per condition. Resting time of thirty seconds were given in-between conditions. A total of seven and a half minutes spent by each student for task completion.

However, in this particular study, only the first three conditions, such as the CKC-1, IKC-2, and CEC-3 data were used due to technical difficulties encountered in IEC-4 which was the last condition to be measured on the experiment conducted. When the signals were extracted on the fNIRS device most of the values recorded were 0.000 due to noise on the signal measurement within the detectors. As the authors strive to identify the possible reasons to better improve this study in the future, the reason behind this could be loosening of Velcro strap that causes cap displacement affecting the optodes’ contact with the region of interests.

**Figure 2:**
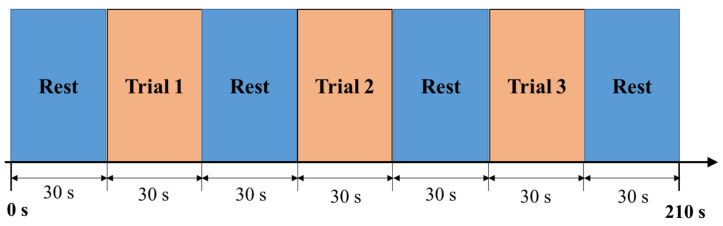
The procedure outline for each task.

Before placing the cap and optodes of the fNIRS system in the subject’s head, the experiment was explained to the students by showing an example of how to select the answer per each condition. Evaluation immediately started with CKC-1, followed by IKC-2, and lastly, CEC-3 after it was made sure that students understood the entire experiment and no questions raised. In each condition, the subjects were asked to indicate the ink color of the color word, not the color word itself. The subjects did this by placing the mouse pointer on the box corresponding to their respective answer. The number of correct answers in each task was documented manually by the researchers using the printed answer key forms to ensure accuracy in checking aside from their familiarity with the subject matter. Only the number of correct responses in self-made CWIT and VR were the focus of this study. However, the response time will be included in future studies. Figure 2 shows the outline of the procedure for the color-word interference test.

### 2.3. FNIRS System and Data Processing

**Figure 3:**
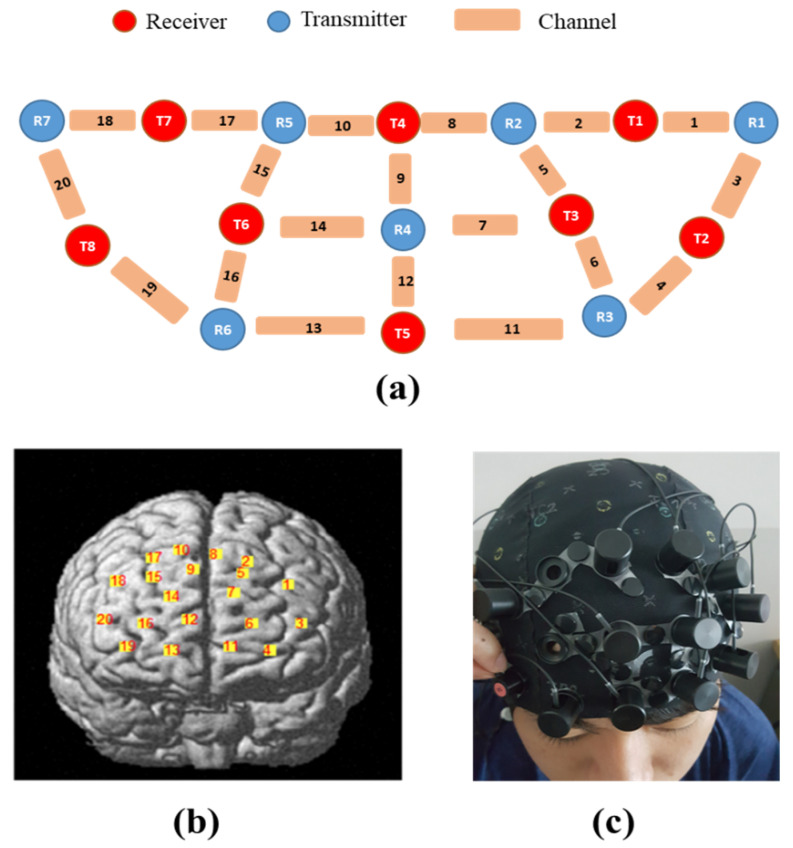
fNIRS system: (**a**) optodes structure, (**b**) channel locations, and (**c**) cap, and optodes.

The measurement of hemodynamic responses through oxy-Hb, deoxy-Hb, and total-Hb was obtained using NIRx NIRSport 2 fNIRS system (NIRx Medizintechnik GmbH, Berlin, Germany). The researchers utilized only the oxy-Hb values as the indicator of regional activation in the brain during task execution, in line with the previous fNIRS studies [1,12], and with the fact that oxy-Hb was proven to be more sensitive than the other parameters [22,23,24,25]. The arrangement of eight sources and seven detectors were able to yield 20 channels. The area covered by the placement of optodes was in the area of the PFC. The placement of optodes structure and fNIRS channels were shown in Figure 3, with the sources shown as red-colored optodes, the detectors as blue-colored optodes, and the channels represented by links from each optode.

The fNIRS signal was converted to concentration changes of hemodynamic parameters using NIRS SPM v4r1 [26], a MATLAB-based software. The parameters set for the conversion were based on the guidelines set on the software. The same software was used to create a mapping of the activated regions of the PFC based on the t-values in each channel from the three tasks [26,27,28,29,30,31].

The wavelet-based filtering and the hemodynamic response function (HRF) were the only utilized during the group analysis using NIRS-SPM, another MATLAB-based software. No other artifact correction was used since the use of HRF reportedly produces the highest increase in contrast-to-noise ratio (CNR) by about 39% [31,32,33,34]. Moreover, this was also in the study of Srikanth and Ramakrishnan [35] in identifying the regions of the brain that are activated through the strength of the amplitude for each task given.

### 2.4. Statistical Analysis

In this study, after extracting the data using MATLAB 2018b the statistical treatments were applied in data using the IBM SPSS version 20. The mean and the linear mixed-effect models (LMMs) for statistical differences across trials and between conditions were used. Specifically, LMMs or the hierarchical or the multi-level or the random effect model is a data analysis method used for repeated measures design. Within the expected results, correlations most likely to exist since the condition of one participant are the same or associated with others who are involved in the study. Moreover, the experimental condition stimuli are presented to all participants [36]. For brain connectivity analysis, the fNIRS device was used.

## 3. Results

### 3.1. What Are the Mean Scores and Overall Mean Scores Obtained in Each Condition per Trial, Both for the Self-Made CWIT and VR?

Table 1 shows the weighted mean and overall mean in test scores and VR. Across all Trials, CKC had the highest mean scores of 91.21 in Trial 1, 92.12 in Trial 2, and 92.73 in Trial 3 with the least VR mean of −0.00018, 0.002, and −0.00012 respectively.

Meanwhile, IKC-2 and CEC-3 were not consistent across all trials. IKC-2 placed second with the higher scores in Trial 2 of 84.85 and CEC-3 higher in Trial 1 with a mean score of 83.94 and in Trial 3 with a mean score of 86.97.

In terms of VR, IKC-2 had the highest VR in Trial 3 only with the mean of 0.00025 while CEC-3 in Trial 1 with the mean of 0.00047 and Trial 2 with the mean of 0.00031.

Overall, the highest CWIT score was recorded in CKC-1 with 92.02, followed by CEC-3 with 85.05, and lastly IKC-2 with 82.73. For VR, the highest was in CEC-3 with 0.00033, next is IKC-2 with 0.00027, and lastly CKC-1 with −0.31.

### 3.2. Are There Any Significant Differences among the Mean Test Scores Obtained per Trial in Each Condition?

Table 2, Table 3 and Table 4 below shows that the hypothesis is rejected that there are no significant differences in test scores in each condition when the tests are repeated. The Tables below show that the *p*-value obtained are all higher than the significant value of <0.05. In particular, it was in CKC-1 were the *p*-value was recorded the highest such as 0.58 and 0.82, followed by IKC-2 with 0.79 and 0.42, and lastly in CEC-3 with the value of 0.32 and 0.37.

### 3.3. Are There Any Significant Differences among the Mean of the VR per Trial in Each Condition?

Table 5, Table 6 and Table 7 below show the significant differences obtained in the means of the VR per trial in each condition and there were no significant differences when the statistical treatment of data was applied thus the hypothesis of this study is rejected. Tables show that the *p*-value per trial in each condition is higher than the significant value of <0.05. CKC-1 had values of 0.77 and 0.12, CEC-3 0.36 and 0.72, and IKC-2 with 0.07 and 0.09.

### 3.4. Are There Any Significant Differences in the Mean Test Scores Obtained per Trial between Congruent and Incongruent Conditions?

Table 8, Table 9 and Table 10 show the significant differences in the mean scores obtained between Congruent and Incongruent Korean Conditions. Across all Trials, findings showed that the hypothesis of this study is accepted that there are significant differences. It is in Trial 3 were *p*-value is the lowest at 0.000, followed by Trial 1 with 0.001, and lastly Trial 2 with 0.003.

### 3.5. Are There Any Significant Differences in the Mean of the VR Obtained per Trial between Congruent and Incongruent Conditions?

Table 11, Table 12 and Table 13 show the significant differences in the mean of the VR obtained between congruent and incongruent Korean conditions. Results showed that it is only in Trial 3 that there was a significant difference between the two thus the hypothesis of the study is accepted with a *p*-value of 0.027, however, rejected in Trial 1 with 0.939 and in Trial 2 with 0.812.

### 3.6. Are There Any Significant Differences in the Mean Test Scores Obtained per Trial between Congruent Conditions?

Table 14, Table 15 and Table 16 shows there are significant differences between the test scores obtained in CKC-1 and CEC-3 thus the hypothesis of this study is accepted with a *p*-value of 0.001. 0.003, and 0.044 respectively.

### 3.7. Are There Any Significant Differences in the Mean of VR per Trial between Congruent Conditions?

Similar to the findings in Section 3.6, Table 17, Table 18 and Table 19 show that it is only in Trial 3 that the hypothesis of this study is accepted with a *p*-value of 0.027. There is a significant difference observed in Trial 3, however no significant difference in Trial 1 with a *p*-value of 0.939 and Trial 2 with a *p*-value of 0.812 between the means of the VR in CKC-1 and CEC-3.

### 3.8. What Are the t-Values Representing the Task Activations on Each Channel?

**Figure 4:**
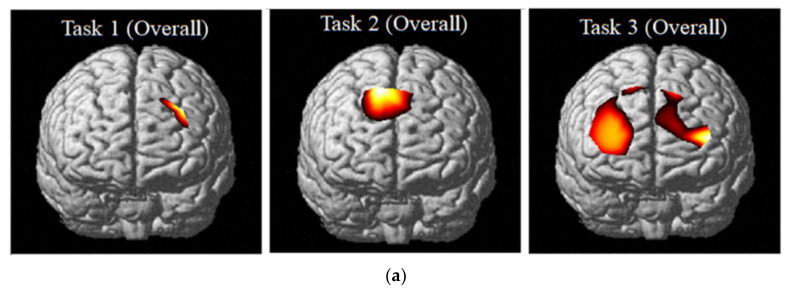

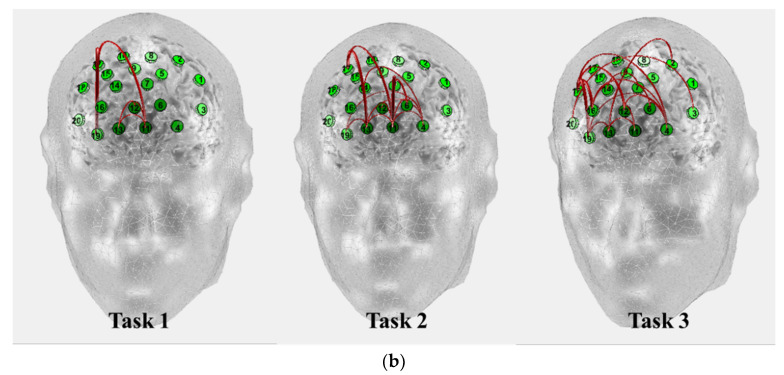
The brain mapping results: (**a**) the activation map, and (**b**) the connectivity map of the prefrontal cortex in each task.

Figure 4a shows the activation map by using NIRS SPM [26] in the PFC across all subjects according to the task done based on the t-values as shown in Table 20. Upon processing for the activation mapping, the level of significance was set to less than 0.05, removing some of the insignificant t-values. The level of significance was based on the comparison of values between each channel.

Generally, CEC-3 can activate left and right prefrontal cortex areas, encompassing the dorsomedial and dorsolateral areas. IKC-2 can activate the superior mid-portion of the prefrontal cortex, which approximately corresponds to the dorsomedial prefrontal cortex (DMPFC) and ACC. CKC activated a small portion of the left DLPFC.

The phase-locking value (PLV) is one of the brain network analysis estimators based on phase synchronization (PS) that identifies transient phase-locking between two neuroelectric signals [37]. If the phase difference varies somewhat across the signals, PLV is close to one; otherwise, it is close to zero. Figure 4b shows the connectivity map by using OptoNet [24]. In the same region as Figure 4a based on PLV.

Upon processing for the brain network mapping, the level of the threshold was set to over 0.95. The PFC hemodynamic network CEC-3 shows strong connectivities in left and right PFC areas, encompassing the dorsomedial and dorsolateral areas. IKC-2 shows the connections to the superior mid-portion of the prefrontal cortex, which approximately corresponds to the DMPFC and ACC. CKC-1 shows similar connections to IKC-2, however less connection and connectivity.

Table 5 shows the *t*-value*s* among the 20 channels during the three Conditions in the CWIT. During CKC-1, channel 1 is noted to have the highest *t*-value, with a *t*-value of 2.97, followed by channel 20 (*t* = 1.68), channel 3 (*t* = 1.46), channel 5 (*t* = 1.38) and channels 14 and 18 (*t* = 1.19). In IKC-2, channel 10 is revealed to have the highest *t*-value of 3.41. Second to channel 10 is channel 9 (*t* = 2.95), followed by channel 8 (*t* = 2.66), channel 14 (*t* = 1.75) and channel 15 (*t* = 1.73). Channel 3 exhibited the highest *t*-value (*t* = 2.72) during ECE-3, followed by channel 16 (*t* = 2.39), channel 2 (*t* = 2.14), channel 10 (*t* = 2.07), and channel 20 (*t* = 1.98). Most of the *t*-value*s* in ECE-3 are above 1.0 compared to the other two conditions.

### 3.9. What Are the Top 10 Networks among fNIRS Channels during the Three Conditions Using the Phase-Locking Value (PLV)?

Table 21 shows the PLV among the top 10 networks among all fNIRS channels during the three Conditions. In CKC, the network of channels 11 to 12 is noted to have the highest PLV of 0.9716, followed by the network of channel 17 to 19 (PLV = 0.9696), and channel 12 to 13 (PLV = 0.9583). In IKC-2, the network of channels 4 to 6 has the highest PLV of 0.9864, followed by the network of channel 4 to 19 (PLV = 0.9846), and channel 11 to 12 (PLV = 0.9816). The network of channels 6 to 16 exhibited the highest PLV of 0.9896 during CEC-3 followed by the network of 4 to 18 (PLV = 0.9870), and channel 13 to 19 (PLV = 0.9868).

## 4. Discussion

The are several available modalities being used nowadays in determining the brain’s activity and in particular, this study focused on the PFC during the administration of self-made CWIT monitored by the fNIRS device to determine if there are any significant differences per trial, between trials in CKC-1 and IKC, between CKC-1 and CEC-3, and across trials using the test scores and VR. Moreover, it is hypothesized in this study that there are significant differences. Aside from the fact that this study focused on the differences present between two sets of tests instead of within- and between-language differences, the authors took the time to analyze brain connectivity.

The conducted experiment revealed that in Table 1 both congruent conditions recorded the highest and higher scores, with overall mean scores of 92.02 and 85.05 than an incongruent condition with an overall score of 82.73. CKC-1 and CEC-3 respectively. This result is supported by the study of Hiroyasu and colleagues [12] where higher correct responses were also attained in the congruent tasks. Moreover, in the study of Bialystok and colleagues [18], it was concluded that the reason for better performance in the congruent task was due to inadvertent reading were both the reading and color-naming have are similar of having the true response. This is supported by the theory of Sterling [10] which is the automaticity that is best to be applied in the congruent conditions since both the ink of the word and the word itself have the same response where control in the attention is no longer needed. Meanwhile, the reason for the incongruent tasks to record the lowest scores is that conflict between color naming was not addressed to get the true response as supported by Bugg and colleagues [19]. Also, the response competition theory of Eriksen and Eriksen [9] supported this for suppression of the word in the incongruent task that must be done to make the response correctly.

Likewise, the result of the native language involving VR in the congruent and incongruent was supported by the study of Schroeter and colleagues [4]. The findings were particularly higher in the IKC-2 with a mean of 0.00027 as compared with CKC-1 with −0.31. It was mentioned that brain activation involved stronger hemodynamic response due to interference presented

In terms of the significant differences in the means of test scores, as shown in Table 2, Table 3 and Table 4 the *p*-value*s* obtained were 0.58 and 0.82, 0.79 and 0.42, and lastly, 0.32 and 0.37 respectively. For VR responses in Table 5, Table 6 and Table 7 per trial in each condition the *p*-value*s* were 0.77 and 0.12, and 0.07 and 0.09, and lastly 0.36 and 0.72. The findings showed that there are no significant differences when statistical treatment data was applied thus the hypothesis in this study is rejected. The reason is that no intervention is provided during rest time such as a review of the test items where the subjects got incorrect responses. As supported by Dweck’s Mindset Theory in which interventions give progressive and likely outcomes as mentioned in the study of Orosz and colleagues [38].

In comparison with the significant difference of mean scores obtained per trial between CKC-1 and IKC-2, the hypothesis is accepted that there are significant differences. Across all trials, differences can be observed in Table 8, Table 9 and Table 10 which scores in CKC-1 of 91.21, 91.12, and 92.73 are higher as compared with IKC-2 test scores of 81.21, 84.85, and 82.12. Aside from the studies of Bialystok and colleagues [18] and Bugg and colleagues [19] to support the findings of the present study, Triesman [8] validated this result that incongruent conditions are difficult than incongruent conditions due to interferences presented, in particular the color itself. On the other hand, for the mean of the VR in Table 11, Table 12 and Table 13, it is only in Trial 3 (Table 13) where significant differences were observed having a *p*-value of 0.027. A significant difference in this trial can be observed for the reason that when conferring with the mean responses as shown in Table 1, the lowest mean of VR was recorded in this Trial across conditions except in IKC-2.

For CKC-1 and CEC-3 as shown in Table 14, Table 15 and Table 16, despite their congruency conditions it is still evident that significant differences can be observed between them having *p*-value*s* of 0.001, 0.003, and 0.000 respectively. These findings are supported by the study of Marian and colleagues [20] where color and language proficiency is expected to be encountered by individuals who are bilinguals. Meanwhile, in terms of the mean of the VR in Table 17, Table 18 and Table 19 with *p*-values of 0.939, 0.812, and 0.027 respectively, it is only in Trial 3 of Table 19 where the hypothesis is accepted. The significant difference is most likely to be observed since brain activation is stronger in the incongruent conditions as compared with the congruent tasks causing the increase in VR as mentioned in the study of Schroeter [4].

In terms of the analysis in the brain connectivity using the PLV that can identify the phase-locking between the two neuro-electric signals [37], CEC-3 showed connectivities in the left and right of dorsomedial and dorsolateral of pre-frontal areas strongly. CKC-1 and IKC-2 were the same but less in CKC-1 due to less brain activation in the congruent conditions as compared with incongruent therefore less VR response [4]. The entire activated parts of PFC can be seen on the figures in this study using the *t*-values shown in Table 20. PFC is activated since it is responsible for executive functions [6] and language based on several studies conducted in the past [1,12,13,14,15,16].

In light of the above findings, it can be concluded that test scores and VR differ between the congruent and incongruent conditions due to interferences encountered. Moreover, in the connectivity analysis, right and left PFC are activated on ECC-3. In CKC-1 and IKC-2, encompassing to dorsomedial and dorsolateral although CKC-1 has less connection and connectivity due to less brain activation as compared.

Aside from color, the language was also considered a factor that must be suppressed in this experiment to give a correct response. Moreover, the *t*-values obtained showed specific parts of the brain that are activated that entirely focused on the PFC. Due to technical difficulties encountered, the data for IEC-4 were not presented. Moreover, response time was not included which the authors plan to address in future studies such as the automatic checking of answers using a developed software with corresponding response time and improved security in cap placement. However, the authors believe the result of this study could be of use as a reference among future researchers in using fNIRS to determine the brain connectivity among patients in a non-invasive way using non-ionizing radiation device without requiring the patient to go to the hospital since it is portable and at low-cost, thus promoting health, comfort, and financial advantages among patients towards a high quality of life.

## 5. Conclusions

In the light of the above findings, fNIRS could be of use as a tool in investigating the brain connectivity and this could improve patient’s quality of life for it is portable, non-invasive, non-iozing, and low-cost. In the experiment conducted, it was shown that the right and left PFC are activated on ECC-3. In CKC-1 and IKC-2, encompassing dorsomedial and dorsolateral although CKC-1 has less connection and connectivity due to less brain activation as compared. The self-made CWIT showed that true response both in the reading and color naming in the congruent conditions cause less brain activation, thus the lowest and lower VR were recorded in the CKC-1 and CEC-3 unlike in the IKC-2. Moreover, language supression was needed between CKC-1 and CEC-3, thus brain was more activated in the CEC-3 with higher VR.

## Figures and Tables

**Table 1:** Comparison of mean scores and mean HbO responses in each trial per task.

Condition	Trial 1		Trial 2		Trial 3		Overall	
	$\bar{x}$ Score	$\bar{x}$ VR	$\bar{x}$ Score	$\bar{x}$ VR	$\bar{x}$ Score	$\bar{x}$ VR	$\bar{x}$ Score	$\bar{x}$ VR
CKC-1	91.21	−0.00018	92.12	0.0002	92.73	−0.00012	92.02	−0.31
IKC-2	81.21	−0.00019	84.85	0.00027	82.12	0.00025	82.73	0.00027
CEC-3	83.94	0.00047	84.24	0.00031	86.97	0.00021	85.05	0.00033

Values displayed were rounded off to its nearest hundredths but calculated using the whole value. VR = vascular response.

**Table 2:** Significant differences among mean scores obtained per trial in CKC-1.

CKC-1	Estimate	Std. Error	df	*t*-Value	*p*-Value
Intercept	92.73	1.90	30	48.92	0.00 *
Trial = 1	−1.52	2.68	30	−0.57	0.58
Trial = 2	−0.61	2.68	30	−0.23	0.82
Trial = 3	0 ^b^	0.00			

^b^ = fixed effect coefficient; df = degrees of freedom; * *p*-value is significant (<0.05).

**Table 3:** Significant differences among mean scores obtained per trial in IKC-2.

IKC-2	Estimate	Std. Error	df	*t*-Value	*p*-Value
Intercept	82.12	2.33	30	35.18	0.00 *
Trial = 1	−0.91	3.30	30	−0.275	0.79
Trial = 2	2.73	3.30	30	0.826	0.42
Trial = 3	0 ^b^	0.00			

^b^ = fixed effect coefficient; df = degrees of freedom; * *p*-value is significant (<0.05).

**Table 4:** Significant differences among mean scores obtained per trial in CEC-3.

CEC-3	Estimate	Std. Error	df	*t*-Value	*p*-Value
Intercept	86.97	2.13	30	40.83	0.00 *
Trial = 1	−3.03	3.01	30	−1	0.32
Trial = 2	−2.73	3.01	30	−0.91	0.37
Trial = 3	0 ^b^	0.00			

^b^ = fixed effect coefficient; df = degrees of freedom; * *p*-value is significant (<0.05).

**Table 5:** Significant differences among the mean of VR obtained per trial in CKC-1.

CKC-1	Estimate	Std. Error	df	*t*-Value	*p*-Value
Intercept	−0.000119	0.000140	30	−0.85	0.40
Trial = 1	−0.000059	0.000198	30	−0.296	0.77
Trial = 2	0.000322	0.000198	30	1.625	0.12
Trial = 3	0 ^b^	0.000000			

^b^ = fixed effect coefficient; df = degrees of freedom.

**Table 6:** Significant differences among the mean of VR obtained per trial in IKC-2.

IKC-2	Estimate	Std. Error	df	*t*-Value	*p*-Value
Intercept	0.000252	0.000165	30	1.53	0.14
Trial = 1	−0.000440	0.000234	30	−1.9	0.07
Trial = 2	0.000019	0.000234	30	0.08	0.09
Trial = 3	0 ^b^	0.000000			

^b^ = fixed effect coefficient; df = degrees of freedom.

**Table 7:** Significant differences among the mean of VR obtained per trial in CEC-3.

CEC-3	Estimate	Std. Error	df	*t*-Value	*p*-Value
Intercept	0.000205	0.000201	30	1.02	0.32
Trial = 1	0.000265	0.000285	30	0.93	0.36
Trial = 2	0.000102	0.000285	30	0.36	0.72
Trial = 3	0 ^b^	0.000000			

^b^ = fixed effect coefficient; df = degrees of freedom.

**Table 8:** The significant difference of mean scores obtained in Trial 1 between CKC-1 and IKC-3.

Trial 1	Estimate	Std. Error	df	*t*-Value	*p*-Value
Intercept	81.21	2.55	10	31.81	0.000 *
CKC-1	10.00	2.11	10	4.74	0.001 *
IKC-3	0 ^b^	0.00			

^b^ = fixed effect coefficient; df = degrees of freedom; * *p*-value is significant (<0.05).

**Table 9:** The significant difference of mean scores obtained in Trial 2 between CKC-1 and IKC-3.

Trial 2	Estimate	Std. Error	df	*t*-Value	*p*-Value
Intercept	84.85	2.03	10	41.83	0.000 *
CKC-1	7.27	1.84	10	3.94	0.003 *
IKC-3	0 ^b^	0.00			

^b^ = fixed effect coefficient; df = degrees of freedom; * *p*-value is significant (<0.05).

**Table 10:** The significant difference of mean scores obtained in Trial 3 between CKC-1 and IKC-3.

Trial 3	Estimate	Std. Error	df	*t*-Value	*p*-Value
Intercept	82.12	2.39	10	34.36	0.00 *
CKC-1	10.61	1.33	10	7.95	0.00 *
IKC-3	0 ^b^	0.00			

^b^ = fixed effect coefficient; df = degrees of freedom; * *p*-value is significant (<0.05).

**Table 11:** The significant difference of the mean of VR obtained in Trial 1 between CKC-1 and IKC-3.

Trial 1	Estimate	Std. Error	df	*t*-Value	*p*-Value
Intercept	−0.000188	0.000097	20.9	−1.94	0.067
CKC-1	−0.000010	0.000127	16.2	0.08	0.939
IKC-3	0 ^b^	0.00			

^b^ = fixed effect coefficient; df = degrees of freedom.

**Table 12:** The significant difference of the mean of VR obtained in Trial 2 between CKC-1 and IKC-3.

Trial 2	Estimate	Std. Error	df	*t*-Value	*p*-Value
Intercept	0.000272	0.000228	16.91	1.19	0.250
CKC-1	−0.000069	0.000286	18.73	0.24	0.812
IKC-3	0 ^b^	0.00			

^b^ = fixed effect coefficient; df = degrees of freedom.

**Table 13:** The significant difference of the mean of VR obtained in Trial 3 between CKC-1 and IKC-3.

Trial 3	Estimate	Std. Error	df	*t*-Value	*p*-Value
Intercept	0.000252	0.000108	13.17	2.33	0.037 *
CKC-1	−0.000371	0.000150	13.03	−2.48	0.027 *
Conditions = 2	0 ^b^	0.00			

^b^ = fixed effect coefficient; df = degrees of freedom; * *p*-value is significant (<0.05).

**Table 14:** The significant difference of mean scores obtained in Trial 1 between CKC-1 and CEC-3.

Trial 1	Estimate	Std. Error	df	*t*-Value	*p*-Value
Intercept	83.94	1.48	10	56.78	0.000 *
CKC-1	7.27	1.55	10	4.71	0.001 *
CEC-3	0 ^b^	0.00			

^b^ = fixed effect coefficient; df = degrees of freedom; * *p*-value is significant (<0.05).

**Table 15:** The significant difference of mean scores obtained in Trial 2 between CKC-1 and CEC-3.

Trial 2	Estimate	Std. Error	df	*t*-Value	*p*-Value
Intercept	84.24	2.16	10	39	0.000 *
CKC-1	7.88	1.97	10	3.99	0.003 *
CEC-3	0 ^b^	0.00			

^b^ = fixed effect coefficient; df = degrees of freedom; * *p*-value is significant (<0.05).

**Table 16:** The significant difference of mean scores obtained in Trial 3 between CKC-1 and CEC-3.

Trial 3	Estimate	Std. Error	df	*t*-Value	*p*-Value
Intercept	86.97	2.60	10	33.45	0.000 *
CKC-1	5.76	2.51	10	2.98	0.044 *
CEC-3	0 ^b^	0.00			

^b^ = fixed effect coefficient; df = degrees of freedom; * *p*-value is significant (<0.05).

**Table 17:** The significant difference of the mean of VR obtained in Trial 1 between CKC-1 and CEC-3.

Trial 1	Estimate	Std. Error	df	*t*-Value	*p*-Value
Intercept	0.000188	0.000097	20.904	−1.936	0.067
CKC-1	0.000010	0.000127	16.201	0.078	0.939
CEC-3	0 ^b^	0.00			

^b^ = fixed effect coefficient; df = degrees of freedom.

**Table 18:** The significant difference of the mean of VR obtained in Trial 2 between CKC-1 and CEC-3.

Trial 2	Estimate	Std. Error	df	*t*-Value	*p*-Value
Intercept	0.000272	0.000228	16.912	1.192	0.250
CKC-1	−0.000069	0.000286	18.733	−0.242	0.812
CEC-3	0 ^b^	0.00			

^b^ = fixed effect coefficient; df = degrees of freedom.

**Table 19:** The significant difference of the mean of VR obtained in Trial 3 between CKC-1 and CEC-3.

Trial 3	Estimate	Std. Error	df	*t*-Value	*p*-Value
Intercept	0.000252	0.000108	13.166	2.327	0.037 *
CKC-1	0.000371	0.000150	13.032	−2.482	0.027 *
CEC-3	0 ^b^	0.00			

^b^ = fixed effect coefficient; df = degrees of freedom; * *p*-value is significant (<0.05).

**Table 20:** *t*-Value*s* representing task activations according to channel.

Channel	Task 1	Task 2	Task 3
**1**	2.97	0.95	0.77
**2**	1.4	1.46	2.14
**3**	1.46	0.72	2.72
**4**	0.48	1.11	1.23
**5**	1.38	1.45	1.81
**6**	0.17	0.23	1.87
**7**	−0.08	1.4	1.93
**8**	1.11	2.66	1.76
**9**	0.62	2.95	1.28
**10**	1.4	3.41	2.07
**11**	0.58	0.56	0.03
**12**	−0.32	1.13	1.43
**13**	−0.12	0.8	1.58
**14**	1.19	1.75	1.69
**15**	0.54	1.73	1.68
**16**	−0.08	0.57	2.39
**17**	0.07	1.53	1.72
**18**	1.19	1.65	1.8
**19**	0.03	0.85	2
**20**	1.68	0.71	1.98
**Total**	**0.78 (±0.80)**	**1.38 (±0.81)**	**1.69 (±0.56)**

**Table 21:** PLVs of top 10 representing task connectivities according to channel connection.

	Task 1		Task 2		Task 3	
	Network	PLV	Network	PLV	Network	PLV
**Top 1**	CH 11-12	0.972	CH 4-6	0.986	CH 6-16	0.990
**Top 2**	CH 17-19	0.970	CH 4-19	0.985	CH 4-18	0.987
**Top 3**	CH 12-13	0.958	CH 11-12	0.982	CH 13-19	0.987
**Top 4**	CH 11-13	0.957	CH 11-17	0.981	CH 7-14	0.985
**Top 5**	CH 11-17	0.952	CH 13-17	0.979	CH 11-12	0.984
**Top 6**	CH 11-19	0.938	CH 4-11	0.979	CH 4-6	0.979
**Top 7**	CH 12-17	0.927	CH 6-11	0.978	CH 3-20	0.979
**Top 8**	CH 12-19	0.925	CH 4-13	0.976	CH 2-13	0.977
**Top 9**	CH 13-17	0.925	CH 7-11	0.975	CH 4-12	0.977
**Top 10**	CH 13-19	0.918	CH 6-7	0.975	CH 4-16	0.976
